# New Onset Diabetes After Organ Transplantation: Risk Factors, Treatment, and Consequences

**DOI:** 10.3390/diagnostics15030284

**Published:** 2025-01-25

**Authors:** Lucija Popović, Tomislav Bulum

**Affiliations:** 1 Department of Emergency Medicine, University Hospital Centre Zagreb, Kišpatićeva 12, 10000 Zagreb, Croatia; 2 School of Medicine, University of Zagreb, Šalata 3, 10000 Zagreb, Croatia; 3 Department of Diabetes and Endocrinology, Vuk Vrhovac University Clinic for Diabetes, Endocrinology and Metabolic Diseases, Merkur University Hospital, Dugi dol 4a, 10000 Zagreb, Croatia

**Keywords:** new onset diabetes after organ transplantation, risk factors, immunosuppressive medication, diabetes

## Abstract

New onset diabetes mellitus after organ transplantation (NODAT) is a frequent and serious complication of solid organ transplantation. It significantly impacts graft function, patient survival, and quality of life. NODAT is diagnosed based on the criteria for type 2 diabetes, with the oral glucose tolerance test (OGTT) serving as the gold standard for diagnosis. The development of NODAT is influenced by a range of risk factors, which are classified into modifiable and non-modifiable categories. Post-transplant, regular glycemic monitoring at specific intervals is essential for timely diagnosis and initiation of therapy. Early intervention can help prevent or delay the onset of diabetes-related complications. The treatment strategy for NODAT involves lifestyle modifications and pharmacological interventions. These include medications such as metformin, sulfonylureas, glinides, thiazolidinediones, DPP-4 inhibitors, GLP-1 agonists, SGLT-2 inhibitors, and insulin. Adjusting immunosuppressive therapy—either by reducing dosages or substituting drugs with lower diabetogenic potential—is a common preventative and therapeutic measure. However, this must be performed cautiously to avoid acute graft rejection, which poses a greater risk to the patient compared to NODAT itself. In addition to managing diabetes, addressing comorbidities such as hypertension and dyslipidemia is crucial, as they elevate the risk of cardiovascular events and mortality. Patients with NODAT are also prone to developing common diabetes-related complications, including diabetic nephropathy, neuropathy, retinopathy, and peripheral vascular disease. Therefore, regular follow-ups and appropriate treatment are vital to maintaining quality of life and improving long-term outcomes.

## 1. Introduction

The progress of medicine in the second half of the 20th century has led to the extremely rapid development and improvement of transplantation medicine. With advancements in immunosuppressive therapy protocols, organ transplantation has become more common, driving progress in postoperative patient care and significantly enhancing their quality of life and survival rates [1]. However, it is important to recognize that organ transplantation and immunosuppression are associated with certain metabolic changes, particularly the risk of developing new-onset diabetes after organ transplantation (NODAT) [2].

NODAT refers to the development of diabetes in patients who did not previously suffer from the condition, following solid organ transplantation. It is a common complication that increases the risks of infections, graft rejection, cardiovascular disease, and overall patient mortality [3,4]. Hyperglycemia, which often arises from the body’s stress response post-transplantation or the use of high-dose corticosteroids to prevent transplant rejection, is observed in most patients during the early postoperative period [5].

]In addition to common risk factors for type 2 diabetes mellitus (T2DM) observed in the general population, organ transplant recipients are continuously exposed to immunosuppressive therapy, which is among the most significant risk factors for developing NODAT. Early diagnosis and the initiation of appropriate therapy are crucial for improving the prognosis of patients with NODAT. Further research is essential to identify preventive measures for NODAT, establish optimal therapeutic strategies, evaluate the impact of glycemic control on graft survival and function, and develop immunosuppressive therapies with minimal diabetogenic effects [1].

## 2. Definition

The literature includes several terms to describe diabetes associated with organ transplantation. NODAT specifically refers to patients diagnosed with diabetes after organ transplantation, excluding those with pre-existing diabetes or those with transient hyperglycemia observed during hospitalization. The latter often results from the body’s stress response or the administration of high-dose corticosteroids [1,6].

Before 2003, the term “post-transplant diabetes” (PTDM) was used to describe only patients requiring insulin therapy 30 days after solid organ transplantation, excluding those with other glucose metabolism disorders [6]. This term was reintroduced in 2014 and now encompasses a broader category, including patients with persistent hyperglycemia, likely caused by undiagnosed diabetes before transplantation, patients with NODAT, and those with transient hyperglycemia lasting up to one year post-transplantation [1].

### 2.1. Etiology and Pathophysiology

The etiology of NODAT is multifactorial, encompassing both non-modifiable and modifiable factors. The non-modifiable factors include age, ethnicity, and genetic predisposition, while modifiable factors consist of obesity, metabolic syndrome, and specific immunosuppressive drug regimens. Older age, along with ethnic groups that have a higher predisposition to type 2 diabetes—such as African American, Hispanic, and Asian populations—significantly increases susceptibility. Additionally, obesity and pre-existing metabolic disturbances heighten the risk by amplifying peripheral insulin resistance. Factors related to the donor organ, as well as post-transplant complications such as cytomegalovirus infections, acute rejection episodes, and subsequent weight gain, also play critical roles in the development of NODAT [6].

The pathophysiology of NODAT is characterized by a combination of factors, including impaired insulin secretion, increased insulin resistance, and damage to pancreatic beta cells. Immunosuppressive therapies significantly contribute to these pathological processes. Calcineurin inhibitors, including tacrolimus and cyclosporine, directly impair beta-cell function by reducing insulin secretion and promoting apoptosis [6,7,8]. Meanwhile, corticosteroids exacerbate insulin resistance by increasing hepatic gluconeogenesis and inhibiting glucose uptake in peripheral tissues [9,10]. Additionally, mTOR inhibitors further worsen insulin resistance by disrupting the insulin signaling pathways in skeletal muscle and adipose tissue [11,12].

The development of NODAT is driven by a multifactorial interplay between non-modifiable factors, such as genetic predisposition, age, and ethnicity, and modifiable factors, such as immunosuppressive therapies and obesity. These factors disrupt glucose homeostasis by inducing beta-cell dysfunction, increasing insulin resistance, and impairing glucose uptake.

### 2.2. Incidence and Prevalence

The incidence of NODAT varies based on the transplanted organ, the immunosuppressive therapy used, the presence of risk factors, and the observation period. Reported incidence rates in the literature range from 2% to 53% among solid organ transplant recipients [1]. NODAT is observed in 10–20% of kidney transplant patients, 20–30% of heart transplant patients, 20–40% of lung transplant patients, and 30–40% of liver transplant patients [13]. In liver transplant patients with hepatitis C virus infection, the incidence increases to 40–60% [8]. NODAT is most commonly diagnosed within the first few months post-transplantation, when patients are administered high doses of immunosuppressive medications. Among kidney transplant recipients, the incidence in the first six months is 20.5%. After this period, the incidence declines and stabilizes at approximately 6% annually, which is comparable to the rate of diabetes in patients on transplant waiting lists [14]. If diabetes is diagnosed later in the post-transplant period, it may represent T2DM rather than NODAT [6].

### 2.3. Diagnosis

Before 2003, post-transplantation diabetes was defined using different criteria. Diagnosis was based on a random blood glucose value > 11.1 mmol/L, a fasting blood glucose value > 7.7 mmol/L, or the requirement for hyperglycemia therapy with oral hypoglycemic agents or insulin following organ transplantation. In 2003, the guidelines for the diagnosis and management of NODAT were established through a consensus of experts in transplant medicine and diabetology. According to the guidelines, the diagnosis of NODAT should be based on the criteria of the American Diabetes Association (ADA) for the diagnosis of T2DM, which include a random glucose ≥ 11.1 mmol/L with symptoms, fasting glucose ≥ 7 mmol/L on more than one occasion, two-hour glucose after an oral glucose tolerance test (OGTT) ≥ 11.1 mmol/L, or glycated hemoglobin (HbA1c) > 6.5%. All patients should undergo OGTT in the pre-transplant evaluation, according to the guidelines of the World Health Organization (WHO), with 75 g of glucose dissolved in water. OGTT allows earlier identification of at-risk patients on the waiting list, and the patients who have an impaired glucose tolerance before transplantation should be screened once a year and change their lifestyle (exercise, weight control, healthy diet), and, if necessary, oral hypoglycemic or insulin therapy should be introduced.

**Figure 1:**
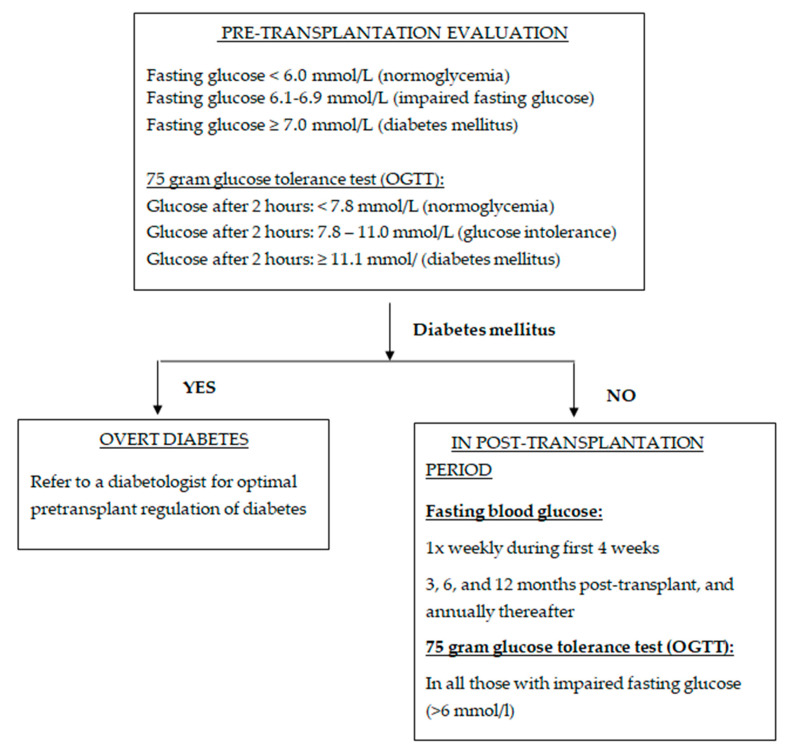
Monitoring glycemia before and after transplantation.

After transplantation, fasting blood glucose levels are monitored at 3, 6, and 12 months post-transplant, and annually thereafter, as the risk of NODAT diminishes over time (Figure 1). Although OGTT is the most sensitive test for detecting glucose metabolism disorders, it is impractical for routine use in all patients [6]. HbA1c, commonly used for diagnosing and monitoring diabetes, is unsuitable for patients on transplant waiting lists or within the first three months post-transplantation. Anemia, which is common during this period, can distort HbA1c results. However, HbA1c becomes reliable for diagnosing and monitoring NODAT beyond three months post-transplant, once blood counts normalize [15,16,17,18,19]. While OGTT remains the gold standard for NODAT diagnosis, fasting blood glucose and HbA1c measurements are effective for identifying high-risk patients requiring further diagnostic evaluation. These measures are also critical for long-term patient monitoring [20].

## 3. Risk Factors

NODAT is a serious complication that arises after solid organ transplantation, significantly increasing patient morbidity and mortality while reducing quality of life. Identifying risk factors, implementing measures for the early detection of high-risk patients, and addressing these risk factors through targeted medical interventions can improve the long-term prognosis of patients with NODAT [20]. The metabolic risk factors involved in the pathogenesis of T2DM similarly contribute to the development of NODAT, compounded by the effects of immunosuppressive therapy. Risk factors for the development of NODAT are categorized into modifiable risk factors, such as immunosuppressive therapy, viral infection, obesity, and insufficient physical activity, and non-modifiable risk factors, such as age, race, genetic predisposition, organ donor characteristics, positive family history of diabetes, previous glucose intolerance, or previous corticosteroid therapy [6]. (Table 1)

### 3.1. Modifiable Risk Factors

#### 3.1.1. Obesity

Most studies have established a connection between obesity and the occurrence of NODAT [21]. Adipose tissue increases the production of tumor necrosis factor-alpha (TNF-α), which disrupts the glucose receptor phosphorylation and reduces the expression of insulin-dependent glucose transporters. This mechanism contributes to the development of insulin resistance and diabetes. Additionally, adipose tissue promotes the synthesis of interleukin-6 (IL-6), which is associated with impaired glucose tolerance. An increase in adipose tissue also leads to decreased secretion of adiponectin, a hormone that helps reduce the risk of diabetes [8]. Low serum adiponectin levels prior to transplantation have been identified as an independent risk factor for NODAT development [22]. The analysis of the USRDS database revealed that obesity, defined as a body mass index (BMI) ≥ 30 kg/m^2^, is a significant risk factor for NODAT (relative risk (RR) 1.73, *p* < 0.0001). In a study of 857 kidney transplant patients, a median follow-up of 5.3 years showed that BMI was a key risk factor for NODAT. The incidence of NODAT was 1.5 per 100 person-years in patients with a BMI < 25 kg/m^2^, 3.3 per 100 person-years in those with a BMI of 25–29.9 kg/m^2^, and 5.6 per 100 person-years in those with a BMI ≥ 30 kg/m^2^ (*p* < 0.0001) [23]. The risk of NODAT increases linearly with each kilogram of body weight above 45 kg [24].

Although some studies have not demonstrated a direct link between obesity and NODAT, obesity and the associated peripheral insulin resistance are widely recognized as significant risk factors for the development of T2DM. The distribution of adipose tissue also plays a significant role in the development of insulin resistance. Studies on healthy women have shown a stronger association between visceral obesity and insulin resistance, compared to the gynoid form of obesity, though similar studies on transplant recipients are lacking [7]. It is suggested that intra-abdominal fat or the waist-to-hip ratio (WHR) may be more significant risk factors for NODAT than total body mass or BMI [16]. Some transplant patients may have been malnourished before transplantation, but the combination of increased hunger, insulin resistance, high-dose corticosteroid use, and insufficient physical activity often leads to rapid weight gain post-transplant. This highlights the importance of lifestyle and dietary changes before transplantation to reduce the risk of NODAT [1].

#### 3.1.2. Metabolic Syndrome

Retrospective studies have shown that the presence of multiple components of the metabolic syndrome increases the risk of developing NODAT [25]. Components of the metabolic syndrome include waist circumference ≥ 102 cm for men or ≥88 cm for women, fasting glucose ≥ 5.6 mmol/L, blood pressure ≥ 130/85 mmHg, fasting triglycerides ≥ 1.7 mmol/L, and HDL cholesterol < 1.04 mmol/L for men or <1.29 mmol/L for women. In a 2010 retrospective analysis of 640 kidney transplant patients, Bayer et al. demonstrated that the prevalence of NODAT at one year increased with the number of metabolic syndrome components. The prevalence was 0% in patients with no metabolic syndrome components, 24% in those with one component, 29% in those with two components, 31% in those with three components, 35% in those with four components, and 74% in patients with all five components of the metabolic syndrome (*p* = 0.001) [26].

#### 3.1.3. Proteinuria

Proteinuria, which typically develops within 3–6 months after organ transplantation, is a risk factor for the development of NODAT. The degree of urinary albumin excretion also plays a significant role: patients with macroalbuminuria have a higher risk compared to those with microalbuminuria, while patients with microalbuminuria have a higher risk than those with normoalbuminuria. The authors of this study suggest that proteinuria could serve as a marker for metabolic syndrome, vascular damage, or both [27].

#### 3.1.4. Hypomagnesemia

Hypomagnesemia is associated with T2DM in the general population, and numerous studies have shown a link between glycemic control and serum magnesium concentrations. Similar results have been observed in liver and kidney transplant patients, where hypomagnesemia was identified as an independent risk factor for developing NODAT [7]. In a retrospective study of 254 kidney transplant patients, Van Laecke et al. demonstrated that hypomagnesemia in the first month after kidney transplantation was associated with the development of NODAT, independent of the immunosuppressive protocol used. Calcineurin inhibitors block the renal magnesium transporter, leading to urinary magnesium loss and subsequent hypomagnesemia. In this study, NODAT resolved after the correction of serum magnesium levels, suggesting that the diabetogenic effect of calcineurin inhibitors is at least partially related to hypomagnesemia [28]. Van Laecke et al. also showed an association between pre-transplantation hypomagnesemia and hypomagnesemia in the first month after transplantation, with the occurrence of NODAT in liver transplant patients [29]. Further studies are needed to determine whether supplementation and correction of serum magnesium concentrations can reduce the incidence of NODAT [7].

#### 3.1.5. Hepatitis C Virus Infection

Infection and inflammation contribute to insulin resistance and diabetes. While the pathogenesis of NODAT associated with hepatitis C virus (HCV) infection is not yet fully understood, it is believed that direct liver cell damage, viral replication in pancreatic beta-cells (leading to their dysfunction and destruction), and the potential effect on the insulin signaling pathway (which affects the synthesis of insulin-related proteins) all play significant roles in the development of insulin resistance, impaired insulin utilization, and insufficient insulin synthesis and secretion [30]. In a cohort study including 17 HCV-positive and 33 HCV-negative orthotopic liver transplant patients, HCV infection was associated with a 62% increase in the incidence of insulin resistance (*p* = 0.0005) [31]. Results from various studies link HCV infection with a 40–60% increase in the incidence of NODAT after liver transplantation [8]. Although interferon is used to treat HCV infection in the general population and has shown effectiveness in glycemic control in organ transplant patients, its use is inadvisable due to its propensity to cause acute graft rejection. Protease inhibitors, which are increasingly used in the general population to treat HCV infection, have not yet been tested in organ transplant patients, and further research is needed [32].

#### 3.1.6. Cytomegalovirus Infection

Kidney transplant patients with symptomatic or asymptomatic cytomegalovirus (CMV) infection are at increased risk of developing NODAT [31]. A study on kidney transplant patients showed a higher incidence of NODAT in those with asymptomatic CMV infection (26%) compared to a healthy control group (6%) [33]. A study by Zielińska et al., which included 276 heart transplant patients, found that patients with CMV infection had a 1.5 times greater risk of developing NODAT (*p* = 0.0179) [20]. It is believed that CMV contributes to reduced insulin secretion through lymphocyte infiltration, increased release of proinflammatory cytokines, and direct damage to pancreatic beta cells, leading to their apoptosis [34].

### 3.2. Non-Modifiable Risk Factors

#### 3.2.1. Age

Age is one of the most significant risk factors for developing NODAT, with the risk increasing by almost 50% for each decade of life [6]. Older age and prolonged dialysis duration are closely associated with an increase in intermuscular adipose tissue and a decline in the Psoas Muscle Index (PMI), both indicators of decreased muscle health. A lower PMI correlates with elevated levels of high-molecular-weight adiponectin, which is associated with insulin resistance and a greater risk of developing NODAT [35]. An analysis of the USRDS database, which included more than 11,000 kidney transplant patients, found a significant association between older age and the development of NODAT. Compared to the reference group of patients aged 18–44 years, those aged 45–59 had a 1.9 times higher relative risk of developing NODAT (*p* < 0.0001), while patients aged ≥ 60 years had a relative risk of 2.09 (*p* < 0.0001) [7]. Many studies consider 45 years as the threshold age for increased risk, though some set this limit at 50 years. Ye et al. concluded that age greater than 50 years increases the risk of developing NODAT in heart transplant patients (HR = 1.20 for age ≥ 50 years vs. age < 50 years, *p* = 0.01) [36]. Similarly, Zielińska et al. found that in a study of 276 heart transplant patients, age greater than 51 years was an independent risk factor for developing NODAT (OR = 1.520, *p* = 0.002) [20].

#### 3.2.2. Race

Numerous studies have shown that African Americans and Latinos are at a higher risk of developing NODAT compared to Caucasian patients, even though most transplant centers worldwide use similar immunosuppressive protocols [37]. The analysis of the USRDS database revealed that NODAT after kidney transplantation is more frequent in African Americans (RR = 1.68, *p* < 0.0001) and Latinos (RR = 1.35, *p* < 0.0001) compared to Caucasians [20]. A study involving 3763 heart transplant patients found that NODAT occurred in more than a quarter of patients during a median follow-up of 2 years. One significant risk factor identified in this study was being of African American race (HR = 0.70 for Caucasians compared to African Americans, *p* < 0.0001) [36]. The influence of race on the occurrence of NODAT is likely related to genetic polymorphisms and the variable effects of immunosuppressive therapy. Tacrolimus, in particular, has been shown to be a more potent cause of NODAT in African American patients compared to Caucasian patients [8]. A meta-analysis of 28 studies on liver transplant patients reported inconclusive results on the effect of race on the occurrence of NODAT in this population, highlighting the need for further research [38].

#### 3.2.3. Genetic Predisposition

The development of NODAT is associated with several genetic factors, including specific human leukocyte antigen (HLA) alleles such as A28, A30, B27, and Bw42. Additionally, multiple studies have identified an association between NODAT and single nucleotide polymorphisms (SNPs), notably the R325W polymorphism in the SLC30A8 zinc transporter gene. Other genetic variants linked to NODAT include KCNQ1, TCF7L2, KCNJ11-Kir6.2, NFATc4, and TCF7L2. These variants are implicated in disrupting insulin secretion and promoting increased gluconeogenesis, processes that contribute to the development of NODAT.

In kidney transplant recipients, the pathogenesis of NODAT has been further associated with genetic variations in the IL-7R, IL-17E, IL-17R, and IL-17RB genes. These cytokines, along with mannose-binding lectin 2 (MBL2), are believed to play a role in the inflammation and damage of pancreatic beta-cells. A study conducted in Malaysia, which included 168 kidney transplant patients, demonstrated a significant association between polymorphisms in the IL-7R gene (HR = 3.15, *p* = 0.01) and the MBL2 gene (HR = 2.57, *p* = 0.04) and the development of NODAT [39]. These findings highlight the important role of genetic predisposition in the onset of NODAT, which may provide valuable insights for the identification of high-risk patients and the development of targeted interventions.

#### 3.2.4. Male Gender

Gender differences play a significant role in the development of NODAT, with men generally having a higher incidence compared to women. These differences are partly attributed to the distinct underlying mechanisms that contribute to NODAT in each gender. In women, pancreatic beta-cell dysfunction appears to be the primary mechanism driving the development of NODAT. This may be related to hormonal differences and how the pancreas responds to the metabolic demands post-transplantation. In contrast, men are more likely to experience insulin resistance and metabolic syndrome as key contributors to the development of NODAT. These gender-specific differences in pathophysiology highlight the complexity of NODAT development and suggest that tailored interventions may be needed for men and women to better manage the risk of NODAT after organ transplantation [40].

#### 3.2.5. Deceased Donor

Patients who receive an organ transplant from a deceased donor have a higher risk of developing NODAT compared to those who receive a transplant from a living donor. This is likely due to various factors, including the longer ischemic time associated with deceased donor organs, which may contribute to organ injury and affect metabolic function. Additionally, the immunological response and the need for more aggressive immunosuppressive therapy in recipients of deceased donor organs might also play a role in the higher incidence of NODAT. A meta-analysis of NODAT incidence after liver transplantation showed that patients receiving a liver from a living donor had a lower incidence of NODAT and a reduced risk compared to those who received a liver from a deceased donor. This difference highlights the potential advantages of living donor transplants, which typically involve shorter cold ischemia times and may result in less organ stress, leading to a reduced risk of complications such as NODAT [7,38].

#### 3.2.6. Positive Family History of Diabetes

Patients with a positive family history of diabetes have an increased risk of developing NODAT, as genetic factors play a significant role in the predisposition to the condition. A study that included 1410 kidney transplant patients, 489 liver transplant patients, 207 heart transplant patients, and 72 lung transplant patients found a strong association between a positive family history of diabetes and a 50% increased risk of developing NODAT in recipients of all types of organs. The odds ratio (OR) for this association was 1.51, indicating a clear genetic influence on the likelihood of developing NODAT in transplant recipients with a family history of diabetes [6,41].

#### 3.2.7. Previous Glucose Intolerance

Impaired glucose tolerance (IGT) is another significant risk factor for the development of NODAT. Patients with pre-diabetes are at an elevated risk of progressing to NODAT, and therefore, they require close monitoring in the post-transplantation period. In addition to regular blood glucose assessments, these patients should receive counseling on lifestyle changes, including recommendations for weight management, healthy eating, and physical activity. These measures can help prevent further weight gain, which is an additional risk factor for the development of diabetes, particularly in the context of post-transplant immunosuppressive therapy [8].

#### 3.2.8. Polycystic Kidney Disease

Autosomal dominant polycystic kidney disease (ADPKD) is the most common inherited cause of kidney failure, and it can lead to significant complications, including the development of NODAT. While some studies have shown an association between ADPKD and NODAT, others have not, prompting further research. A meta-analysis, which included 12 studies and 1379 kidney transplant patients with ADPKD out of a total of 9849 patients, found that the relative risk for developing NODAT in patients with ADPKD was 1.92, compared to those who received a kidney transplant for other reasons. The exact mechanism by which ADPKD leads to NODAT remains unclear, but mutations in the PKD1 and PKD2 genes, which are associated with the disease, may contribute to insulin resistance, impaired insulin secretion, and gluconeogenesis [42].

## 4. Immunosuppressive Medication

**Figure 2:**
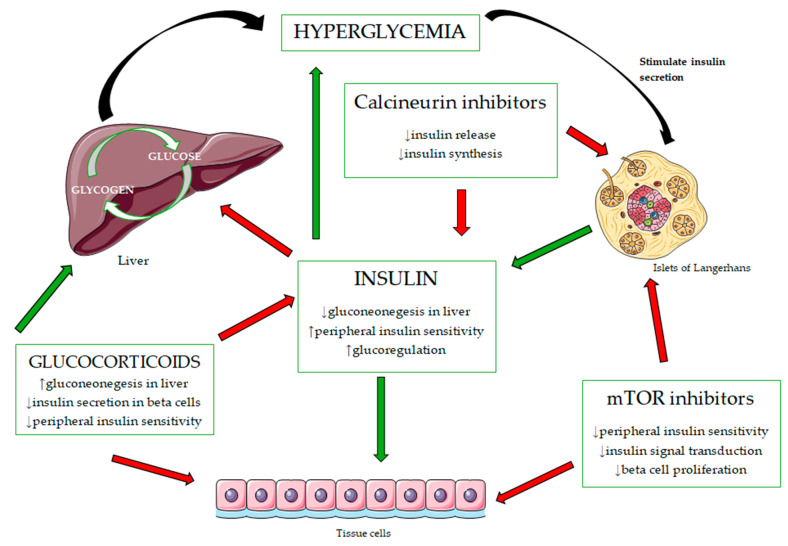
The diabetogenic effect of immunosuppressants (↑ increase, ↓ decrease; green arrows stimulate, red arrows suppress).

The survival of transplant patients has significantly improved due to the development of more effective immunosuppressive therapies. However, despite advancements in immunosuppressive protocols, long-term graft survival has remained relatively unchanged. This is largely due to challenges in effectively managing alloreactive antibodies, as well as the metabolic and cardiovascular consequences of immunosuppressive treatment [43]. One of the main causes of high mortality in transplant patients with functioning grafts is cardiovascular complications, which are often a result of the medications used to prevent graft rejection. Corticosteroids, calcineurin inhibitors, and sirolimus, commonly prescribed as immunosuppressive agents, can lead to hypertension, NODAT, and dyslipidemia (Figure 2). To mitigate the metabolic side effects, antibodies targeting T-lymphocytes are increasingly used during induction therapy. However, these antibodies come with the downside of a higher risk of opportunistic infections and malignancies [43]. In response to these risks, a strategy of combining medications with different mechanisms of action is used, allowing for the administration of the lowest effective dose of each drug and minimizing side effects. Nevertheless, inadequate immunosuppression can lead to graft rejection and early chronic graft dysfunction. In such cases, higher doses of corticosteroids are often required, which can accelerate the development of atherosclerosis and initiate a detrimental cycle of adverse effects [44]. Given these complexities, an individualized approach is essential for selecting the optimal immunosuppressive therapy for each patient. This approach aims to minimize complications while ensuring graft survival and preventing rejection [43,44].

### 4.1. Calcineurin Inhibitors

Calcineurin inhibitors (CNIs), such as tacrolimus and cyclosporine, are essential immunosuppressive medications used to prevent graft rejection in transplant patients. However, calcineurin is also present in pancreatic beta-cells, where it plays a critical role in their proliferation, maturation, and function. CNIs contribute to the development of NODAT by reducing insulin secretion, increasing insulin resistance, and having a toxic effect on pancreatic beta-cells [11,33,45]. Moreover, CNIs induce peripheral insulin resistance and promote hyperglycemia by downregulating the genes that enhance insulin sensitivity in muscles and adipocytes and by reducing the number of GLUT-4 transporters in the membranes of muscle and fat cells [12]. Their diabetogenic effect is exacerbated when used in conjunction with high doses of corticosteroids [46]. The mechanism behind their immunosuppressive action involves binding to intracellular proteins called immunophilins, leading to the inhibition of calcineurin. This results in reduced activation of T-lymphocytes, which in turn lowers the immune response to antigens and significantly reduces graft rejection [47]. Tacrolimus, in particular, is 100 times more potent than cyclosporine in preventing acute graft rejection and improving graft survival. However, tacrolimus is more strongly associated with the development of NODAT compared to cyclosporine [47].

In a large study of 14,452 heart transplant patients, tacrolimus was found to have the highest association with the development of NODAT (HR = 1.459), followed by rifampicin and cyclosporine [48]. Cyclosporine, in contrast to tacrolimus, also inhibits HCV replication, making it a better choice for HCV-positive patients, where tacrolimus may increase the risk of diabetes [49]. Tacrolimus is commonly used as first-line therapy in immunosuppressive protocols and is often combined with other drugs, such as mTOR inhibitors, mycophenolate mofetil, or azathioprine to reduce dosages and minimize toxicity. The most important side effects of CNIs include nephrotoxicity, hypertension, dyslipidemia, NODAT, and increased cardiovascular risk. Nephrotoxicity, which can be acute or chronic, is more pronounced with cyclosporine compared to tacrolimus. Studies in heart and lung transplant patients indicate that tacrolimus is also associated with neurotoxicity, and gastrointestinal toxicity, suggesting the need for further studies to optimize therapy and reduce these toxic effects [50,51,52].

### 4.2. Corticosteroids

Corticosteroids remain a critical component of immunosuppressive therapy, particularly in the early post-transplantation period and during acute graft rejection episodes. The most commonly used corticosteroids in transplant protocols include hydrocortisone, prednisolone, prednisone, and methylprednisolone. While corticosteroids are vital for preventing graft rejection, their long-term use is associated with numerous adverse effects. As a result, the goal is to reduce or discontinue corticosteroids within 3 to 6 months post-transplant, except in cases where low-dose prednisolone is used for maintenance therapy following a graft rejection episode [47]. However, some studies suggest that early discontinuation of corticosteroids may have more adverse effects than benefits, increasing the risk of acute graft rejection, particularly in kidney transplant patients who may require higher doses and prolonged corticosteroid use due to their elevated risk of graft rejection [1]. This risk is particularly concerning for older transplant recipients (≥55 years), as they are more likely to experience acute rejection when corticosteroids are discontinued early [53].

A large retrospective study of over 25,000 kidney transplant patients indicated that protocols excluding corticosteroids were associated with a reduced risk of developing NODAT. Specifically, protocols involving corticosteroids at the time of discharge were linked to a 42% higher risk of developing NODAT [54]. Corticosteroids exert their immunosuppressive effects by suppressing complement binding and antibodies, enhancing IL-10 expression, and reducing IL-2, IL-6, and interferon γ expression in T-lymphocytes [47]. However, corticosteroids also have significant diabetogenic effects, contributing to NODAT development by increasing peripheral insulin resistance, reducing insulin secretion from the pancreas, stimulating gluconeogenesis in the liver, and promoting pancreatic beta-cell apoptosis at high doses [8]. While hyperglycemia may occur shortly after high doses of intravenous methylprednisolone, it is typically transient but may signal an increased risk of future NODAT [46]. In addition to insulin resistance and diabetes, corticosteroids are associated with other side effects, including hyperlipidemia, hypertension, obesity, osteoporosis, and an elevated cardiovascular risk [43].

The extent of corticosteroid-induced NODAT is dose- and duration-dependent. Maintenance immunosuppressive protocols that avoid corticosteroids significantly reduce the risk of NODAT. Even reducing the daily dose of prednisolone to 5 mg improves glucose tolerance. Conversely, an increase in prednisolone dose by just 0.01 mg/kg/day is associated with a 5% higher risk of NODAT [8,33]. High doses of corticosteroids (≥10 mg) are particularly linked to the development of insulin resistance [55]. Short-term corticosteroid pulse therapy post-transplant, combined with low-dose corticosteroids for maintenance, has been found to reduce the risk of developing NODAT [56]. In summary, while corticosteroids are crucial for preventing graft rejection, careful management of their dose and duration is essential to minimize the risk of NODAT and other metabolic complications in transplant patients.

### 4.3. Mammalian Target of Rapamycin (mTOR) Inhibitors

Sirolimus is a mammalian target of rapamycin (mTOR) inhibitor that is structurally similar to tacrolimus. However, while both drugs target intracellular proteins to achieve immunosuppressive effects, their mechanisms of action differ. Unlike tacrolimus, which inhibits calcineurin, sirolimus forms a complex with another intracellular protein, inhibiting the activation of mTOR serine-threonine kinase. This disruption in the IL-2 receptor signaling pathway impairs the proliferation of B and T lymphocytes, reducing the immune response. Sirolimus has antiproliferative properties and was initially thought to reduce interstitial fibrosis, thus potentially extending graft survival [43,47]. However, studies have shown that while sirolimus might help prevent fibrosis, it does not improve graft survival when used alone or in combination with a CNI. In fact, this combination increases the risk of acute rejection, nephrotoxicity, NODAT, dyslipidemia, and thrombotic microangiopathy [43,47]. Specifically, the combination of sirolimus and a calcineurin inhibitor (such as tacrolimus) has a more pronounced diabetogenic effect than CNI monotherapy, leading to a 1.9-fold increased risk of NODAT [57].

The exact mechanism by which sirolimus and other mTOR inhibitors contribute to hyperglycemia remains unclear, but it is generally thought that these drugs increase insulin resistance by disrupting cellular signaling pathways. Additionally, sirolimus directly affects pancreatic beta-cells, reducing insulin secretion, and promotes gluconeogenesis in the liver [46]. Interestingly, a recent meta-analysis comparing the combination of CNIs and mTOR inhibitors in new kidney transplant patients with CNIs and antiproliferative agents showed no significant increase in NODAT incidence after one year of treatment [58]. Similarly, the TRANSFORM study, which compared everolimus (an mTOR inhibitor) with reduced-exposure CNIs against mycophenolate with standard-exposure CNIs, found no significant difference in NODAT incidence between the two groups [59]. This lack of variance could be attributed to the reduced dosages of CNIs used in these studies [60]. While sirolimus is still used in some transplant protocols, its use has become less common due to its higher association with NODAT, acute graft rejection, and other adverse effects. It is now primarily considered a second-line therapy, mainly for patients who cannot tolerate CNIs [47]. Additionally, sirolimus has been associated with an increased risk of hepatic vein thrombosis in liver transplant patients, which further limits its use in this population [47].

### 4.4. Immunosuppressive Medication Associated with a Low Risk of New Onset Diabetes After Organ Transplantation

#### 4.4.1. Antimetabolites

Antimetabolites such as azathioprine and mycophenolate mofetil (MMF) are commonly used in transplant immunosuppressive regimens to inhibit cell proliferation, particularly targeting B and T lymphocytes. These drugs work by inhibiting the synthesis of purines and pyrimidines, which are essential for DNA and RNA synthesis. Specifically, azathioprine and MMF inhibit inosine monophosphate dehydrogenase, an enzyme required for the formation of guanosine monophosphate (GMP), a purine nucleotide necessary for cell replication. While most cells can synthesize GMP via alternative pathways, lymphocytes lack the key enzyme for this, which means that these drugs effectively suppress lymphocyte proliferation, thereby limiting immune response and graft rejection [8,47]. Importantly, neither azathioprine nor MMF has a diabetogenic effect, which makes them a favorable choice in immunosuppressive therapy, especially for minimizing the risk of NODAT. Moreover, MMF has been shown to reduce the diabetogenic effects of tacrolimus, a CNI that is associated with a higher risk of NODAT [47]. The use of antimetabolites such as MMF in combination with other immunosuppressive agents allows for the use of lower doses of more diabetogenic medications such as tacrolimus and corticosteroids, thereby reducing their associated side effects, including the risk of NODAT [8,47]. In summary, azathioprine and MMF are valuable in transplant regimens, not only for their immunosuppressive effects but also for their ability to mitigate the adverse metabolic consequences of other immunosuppressive therapies, such as CNIs.

#### 4.4.2. Belatacept

Belatacept is an immunosuppressive agent that acts by selectively inhibiting T-cell activation. It is a fusion protein composed of a modified extracellular domain of the CTLA-4 antigen (cytotoxic T-lymphocyte antigen 4) and a portion of the Fc fragment of immunoglobulin G1. Belatacept works by binding to the CD80 and CD86 transmembrane glycoproteins on antigen-presenting cells, blocking their interaction with the CD28 glycoprotein on T lymphocytes. This prevents the costimulation required for T-lymphocyte activation and immune response, thus reducing graft rejection [43]. In kidney transplant patients, studies have shown that belatacept provides several benefits compared to CNIs. These advantages include better blood pressure control, improved lipid profiles, enhanced renal function, and a lower incidence of NODAT [43]. The use of belatacept may therefore offer a metabolic benefit by reducing the risk of NODAT, which is a significant concern with CNIs such as tacrolimus and cyclosporine [43,61].

However, one drawback of belatacept is that it is associated with higher rates and severity of acute graft rejection compared to CNIs. This makes it more suitable for patients at low immunological risk, where the risk of rejection is lower. In medically complex patients with a higher risk of graft rejection, a combination of belatacept and low-dose tacrolimus therapy is considered a safer approach. This combination can help reduce the risk of acute rejection while maintaining the metabolic benefits offered by belatacept [43,62,63]. In summary, belatacept represents a promising alternative to CNIs and may mitigate the diabetogenic effects when used in combination with CNIs in specific transplant patient populations, offering advantages in terms of metabolic outcomes and kidney function, while requiring careful management of rejection risks.

## 5. Management and Treatment

The treatment of NODAT requires an individualized approach, taking into account the patient’s characteristics, blood glucose levels, and the impact of immunosuppressive therapy. There is no standard treatment model for NODAT, and guidelines for T2DM are generally applied, with adjustments made based on the specific circumstances of transplant recipients [1]. The glycemic targets for patients with NODAT is to achieve similar targets as those for T2DM: fasting plasma glucose < 6.1 mmol/L, 2 h postprandial plasma glucose < 7.8 mmol/L, and HbA1c < 6.5% [7]. However, in transplant recipients, especially those with other comorbidities, target HbA1c values between 7.5% and 8% may be more appropriate, considering the potential risks of overly aggressive glucose control [64].

Early detection and treatment of hyperglycemia are crucial for improving the long-term survival of both the transplanted organ and the patient, as well as enhancing quality of life. Hyperglycemia in the post-transplant period may result from postoperative stress, pain, and high doses of immunosuppressive medications. In such cases, the first step is to adjust immunosuppressive therapy by reducing the dose or replacing medications with a less diabetogenic effect. However, caution must be exercised to avoid graft rejection, which may require higher doses of immunosuppressants and increase the risk of NODAT [8,46]. Studies show that patients with lower blood glucose levels immediately after transplantation have reduced risk of infection, graft rejection, NODAT, re-hospitalization, and improved long-term outcomes. However, hypoglycemia may limit the benefits of tight glycemic control [64,65]. The main objective is to prevent microvascular and macrovascular complications of diabetes that can impair graft function and patient survival.

The first line of treatment involves adjusting immunosuppressive therapy and encouraging lifestyle changes, such as diet modifications and increased physical activity. If satisfactory glycemic control is not achieved, oral hypoglycemic medications or insulin may be introduced. The choice between insulin and oral hypoglycemic agents depends on the severity of hyperglycemia. In the immediate post-transplant period, intravenous insulin is the preferred option, as it allows for the precise control of glucose levels. It is used until the function of the transplanted organ stabilizes. Once the patient is discharged, oral hypoglycemics can be considered, particularly for patients who only required low doses of insulin during hospitalization. However, renal function should be closely monitored, as impaired kidney function may affect the choice of oral hypoglycemic agents [8,46,65]. For patients who had diabetes before transplantation, their pre-transplant therapy should be resumed if glycemic control was satisfactory. If diabetes was poorly controlled before transplantation, adjustments should be made. It is also important to monitor kidney function, particularly in kidney transplant recipients, as this may influence therapy choices. For patients who did not have diabetes before transplantation but experienced stress-induced hyperglycemia, education on the risk of developing NODAT is essential. Patients should be counseled on lifestyle modifications to reduce this risk [64,65]. Managing NODAT requires a tailored approach, balancing immunosuppressive therapy adjustments with glycemic control. Early intervention and personalized care can improve both transplant outcomes and the patient’s overall health.

### 5.1. Non-Pharmacological Measures

Lifestyle changes reduce the risk of developing NODAT in patients with an increased risk and are supposed to be the first line of treatment for patients with impaired glucose tolerance and NODAT. Patient education plays a crucial role in the implementation of non-pharmacological measures. A nutrition rich in omega-3 and omega-9 fatty acids, complex carbohydrates and fibers manifests a beneficial effect on glycemic regulation. Cessation of smoking, exercising at least 150 min a week and reducing body weight, specifically targeting obese patients, also showed a beneficial effect. Obese patients are advised to lose at least 7% of their initial body weight. Exercise aimed at building muscle mass and reducing fat tissue is potentially more effective than weight reduction alone [33,66]. A diet containing few vegetables and fruit is a predisposing factor for T2DM in the general population. Studies on kidney transplant patients showed a lower risk of NODAT related to vegetable consumption, but not fruit consumption. The relation between NODAT and vegetable consumption was mediated by the effect on the main components of metabolic syndrome, smaller waist circumference, higher HDL cholesterol and lower triglycerides [67,68]. Skeletal muscles have a crucial role in glucose homeostasis and are responsible for 80% of postprandial insulin-stimulated glucose uptake. Insulin resistance disrupts the amount and the time of glucose uptake in skeletal muscles [69]. Daily-life moderate-to-vigorous physical activity (MVPA) is associated with a lower risk of NODAT, cardiovascular mortality and all-cause mortality in kidney transplant patients, compared to non-MVPA patients [70]. A study on kidney transplant patients demonstrates the importance of active lifestyle modification compared to passive intervention. The active intervention group received lifestyle modifications led by a renal dietitian and behavioral therapy intervention, while the passive intervention group received only a leaflet with advice on lifestyle changes and counselling about the risk of NODAT. Active lifestyle intervention resulted in a clinically significant reduction in the incidence of NODAT, improvement in weight and reduction in fat mass; however, it was not associated with a change in insulin secretion and sensitivity [71]. There is evidence that bariatric surgery is beneficial for morbidly obese patients before and after kidney transplantation, improving the access to transplantation for morbidly obese transplant candidates and resulting in similar maintenance of weight loss and improved allograft survival [72,73].

### 5.2. Pharmacological Measures

Pharmacological measures are the second line of therapy for most patients. If the target values of hemoglobin A1c are not achieved by changing lifestyle habits, then monotherapy with oral hypoglycemics is usually started. Combined therapy with two or more oral hypoglycemics or a glucagon-like peptide 1 (GLP-1) analogue is introduced if monotherapy is insufficient to achieve satisfactory glycemia, and if combined therapy does not give satisfactory results, insulin is introduced. Metformin is the first oral hypoglycemic introduced to patients with T2DM, but due to limitations in the use of metformin in patients with renal failure, sulfonylureas are often used as initial therapy. However, recent studies show that metformin is safe in patients with chronic kidney disease stage 3, making it a reasonable choice for first-line treatment in patients with NODAT as well. In addition to sulfonylurea derivatives and metformin, α-glucosidase inhibitors, thiazolidinediones, glinides, GLP-1 receptor agonists, dipeptidyl peptidase 4 (DPP-4) inhibitors, and sodium and glucose co-transporter 2 (SGLT-2) inhibitors are also used. The choice of medication is individualized and adapted to each patient depending on his comorbidities, potential benefits, and the applied immunosuppressive therapy to minimize adverse effects and optimize outcomes [60,64] (Table 2).

#### 5.2.1. Metformin

Metformin, a member of the biguanide class of drugs, is the first-line therapy for patients with T2DM and is also commonly used in patients with NODAT. Metformin works primarily by increasing insulin sensitivity and reducing gluconeogenesis in the liver, helping to control blood sugar levels without causing hypoglycemia. It has an advantage over other hypoglycemic agents because it is not metabolized by cytochrome P450 enzymes, and thus does not interact with most immunosuppressive medications. However, metformin has a significant limitation in patients with impaired kidney function. Since it is excreted by the kidneys, its use in patients with kidney dysfunction increases the risk of lactic acidosis, a potentially fatal condition. This is why its use is generally contraindicated in individuals with serum creatinine levels above 1.5 mg/dL for men and 1.4 mg/dL for women, as per FDA guidelines. Despite this, studies involving patients with a GFR between 30 and 60 mL/min/1.73 m^2^ have demonstrated that lactic acidosis is rare in these patients when metformin is continued under medical supervision [64,74,75]. Newer guidelines recommend adjusting metformin use based on the estimated GFR (eGFR). For patients with an eGFR above 45 mL/min/m^2^, the full dose of metformin can be prescribed. For those with an eGFR between 44 and 30 mL/min/m^2^, the dose should be reduced to a maximum of 1000 mg per day. Metformin should be stopped completely if the eGFR falls below 30 mL/min/m^2^ or in the presence of conditions such as hypoxia or acute kidney function decline (e.g., from sepsis, hypotension, or nephrotoxic medications), which increase the risk of lactic acidosis [74,76].

Metformin is particularly useful in transplant patients who gain weight due to the use of glucocorticoids. In such cases, metformin can promote weight loss, which helps manage the additional metabolic challenges caused by corticosteroids. Additionally, some studies have suggested that metformin may lower the risk of death-censored graft failure, though there has been no significant association with acute rejection or all-cause mortality, as long as lactic acidosis does not occur [77]. Metformin is a valuable medication for managing NODAT in transplant patients, particularly those with preserved kidney function. It helps manage blood glucose levels, avoid hypoglycemia, and even reduce weight gain caused by corticosteroid therapy. However, its use requires careful monitoring of kidney function, particularly given the risk of lactic acidosis in patients with renal impairment.

#### 5.2.2. Sulfonylurea Derivatives and Glinides

Sulfonylurea derivatives and glinides increase insulin secretion from the pancreatic beta-cells, but, over time, exhaustion of the pancreas weakens their effect. Sulfonylurea derivatives and glinides can cause hypoglycemia and weight gain. They are often used in patients with NODAT as oral hypoglycemic agents with the strongest impact on reducing HbaA1c. Giclazide, a newer long-acting sulfonylurea derivative, can also be safely used in patients with chronic kidney disease. Gliclazide is a safer and more effective choice for managing NODAT or T2DM in patients at risk of hypoglycemia or those with renal impairment. Its modern formulation and controlled pharmacodynamics make it a superior alternative to older sulfonylureas such as glyburide and glimepiride. Glyburide and glimepiride also interact with the metabolism of ciclosporin and lead to a significant increase in its concentration. Nateglinide and repaglinide belong to the glinide group and cause a sudden and short-term increase in insulin secretion and therefore should be taken before meals. Nateglinide should be avoided in patients with eGFR < 60 mL/min/m^2^ because of accumulation of its active metabolite, while repaglinide is safe for use in patients with chronic kidney disease and has been successfully used in patients with NODAT and kidney transplants. Cyclosporine and itraconazole can increase the concentration of repaglinide because they inhibit the CYP3A4 enzyme that metabolizes it, thereby increasing the risk of hypoglycemia, but repaglinide does not increase the concentration of cyclosporine, tacrolimus, and sirolimus [64,78].

#### 5.2.3. Inhibitors of α-Glucosidase

Acarbose and miglitol slow the breakdown of oligosaccharides in the small intestine, consequently delaying glucose absorption after a meal without causing hypoglycemia. Their use should be avoided in patients with a creatinine level above 2 mg/dL, and there are limited data on their use in solid organ transplant recipients [64].

#### 5.2.4. Thiazolidinediones

Pioglitazone increases insulin sensitivity and does not cause hypoglycemia. Its primary advantage is that it is metabolized in the liver, making it suitable for patients with chronic kidney disease without dose adjustment. However, a significant side effect is fluid retention, which can exacerbate heart failure, limiting its use in patients with severe liver and kidney disease. Additional caution is required in patients with impaired graft function. Pioglitazone can also cause bone loss, fractures, and weight gain. Pioglitazone has been successfully used in liver and kidney transplant patients and does not affect the metabolism of ciclosporin or tacrolimus [64,79]. In heart transplant patients, pioglitazone is not recommended due to the increased risk of heart failure and the lack of studies proving its safety in this population [80].

#### 5.2.5. Dipeptidyl Peptidase-4 Inhibitors

Dipeptidyl peptidase-4 (DPP-4) inhibitors inhibit the DPP-4 enzyme, thereby preventing the breakdown of glucagon-like peptide 1 (GLP-1), which consequently increases insulin secretion and decreases glucagon secretion from the pancreas. They do not cause hypoglycemia, do not lead to weight gain, and are well tolerated. Sitagliptin, saxagliptin, and alogliptin require dose adjustments in patients with chronic kidney disease. Linagliptin, however, does not require dose reduction in such patients and can be safely used in those with moderate liver disease. With saxagliptin, it is necessary to adjust the dose of ciclosporin and other medications that inhibit CYP3A4, such as itraconazole. Several studies have demonstrated the efficacy and safety of sitagliptin and linagliptin, with no impact on serum concentrations of ciclosporin, tacrolimus, and sirolimus in kidney transplant patients [64,81]. Vildagliptin and sitagliptin are effective and safe options for heart or kidney transplant patients, but vildagliptin should be avoided in patients with impaired liver function or stage IV and V chronic kidney disease, while the dose of sitagliptin should be adjusted for renal insufficiency [7,80]. A study on kidney transplant patients found that initiating sitagliptin for hyperglycemia during the first week after transplantation and discontinuing it by the third month if diabetes does not develop reduces the absolute risk of abnormal OGTT findings by 18% [82].

#### 5.2.6. Glucagon-like Peptide 1 (GLP-1) Receptor Agonists

GLP-1 receptor agonists stimulate insulin secretion, reduce glucagon secretion from the pancreas, slow gastric emptying, and suppress appetite by promoting a feeling of satiety. They lead to weight loss, do not cause hypoglycemia, and improve cardiovascular risk. This group includes exenatide, liraglutide, lixisenatide, albiglutide, semaglutide, and dulaglutide. These medications are primarily administered subcutaneously, with semaglutide being the only member available in an oral formulation. The clearance of exenatide decreases with reduced glomerular filtration, and cases of acute kidney injury related to its use have been reported. Therefore, exenatide is contraindicated in patients with a GFR < 30 mL/min/m^2^. Liraglutide is not metabolized by the kidneys, and no dose adjustment is required for patients with impaired renal function, including those with end-stage renal disease, although data on its use in this population remain limited. Semaglutide and dulaglutide can also be used in patients with impaired kidney function, with dulaglutide being approved for use down to an eGFR of 15 mL/min/m^2^, while semaglutide is contraindicated only in terminal kidney failure [64,83,84].

A retrospective study involving kidney, liver, and heart transplant recipients found that GLP-1 receptor agonists do not impact tacrolimus levels or transplant outcomes over a 12-month follow-up period. These medications also appear to be effective for weight loss and glycemic control, similar to their effects in non-transplant patients with diabetes [85,86]. The largest study to date, involving 118 kidney, liver, and lung transplant patients, confirmed that GLP-1 receptor agonists are both safe and effective in solid organ transplant recipients. Significant improvements were observed in weight loss, HbA1c, and fasting blood glucose levels [87].

#### 5.2.7. Sodium-Glucose Cotransporter-2 (SGLT2) Inhibitors

SGLT-2 inhibitors reduce glucose reabsorption in the proximal tubules of the kidneys, leading to increased glucosuria and a reduction in HbA1c by approximately 0.8%. These medications promote weight loss, do not cause hypoglycemia, and exhibit cardioprotective and renoprotective effects in patients with native kidney disease. However, they are associated with an increased risk of developing urinary tract infections and can cause volume depletion. Data on the use of SGLT-2 inhibitors in kidney transplant patients are currently limited, and larger trials are needed before their routine use in transplant patients can be recommended. Although some studies report benefits consistent with findings in the non-transplant population and a similar frequency of adverse effects compared to transplant patients not receiving SGLT-2 inhibitors, caution is advised, given the limited data available [64,88,89,90,91]. A pharmacokinetic study showed no clinically significant interactions between canagliflozin and ciclosporin. Additionally, a survey of organ transplant patients reported significant reductions in body weight, BMI, systolic and diastolic blood pressure, and the required dose of furosemide in heart transplant patients on empagliflozin therapy, with no significant adverse effects [80]. In a study of 24 kidney transplant patients with diabetes treated with canagliflozin, reductions in body weight, blood pressure, and HbA1c levels were observed, along with a decreased need for other oral hypoglycemics. Importantly, no cases of hypoglycemia or other serious side effects were reported [92].

#### 5.2.8. Insulin

Approximately 50% of patients with NODAT require insulin therapy. The dose and type of insulin are tailored to each individual, as insulin requirements depend on factors such as diet, physical activity, renal function, and the dosage of immunosuppressive medications [46]. Basal insulin can be introduced for patients whose glycemic control remains unsatisfactory despite using two or more oral hypoglycemics. If glycemic targets are still unmet, a combination of basal and bolus insulin is implemented. All forms of insulin can be safely used in organ transplant patients with chronic kidney disease or liver dysfunction, without dose adjustments. However, patients with impaired renal function are at a higher risk of hypoglycemia due to the prolonged half-life of insulin. A study involving patients with an eGFR < 45 mL/min/m^2^ found that those receiving 0.25 units/kg of insulin achieved comparable glycemic control but experienced significantly fewer hypoglycemic episodes than those receiving 0.5 units/kg. Insulin-treated patients must closely monitor their blood glucose levels and adjust the doses to mitigate the risk of hypoglycemia [64,93]. A study on kidney transplant recipients comparing long-acting insulin and intermediate-acting insulin revealed that long-acting insulin glargine provided better blood sugar control but was associated with more frequent hypoglycemic episodes, particularly at night [94].

Despite the extensive range of hypoglycemic agents available for diabetes management and the substantial body of research on T2DM, there is limited clinical data on their use, efficacy, and safety in organ transplant patients. Concerns persist regarding the safety of older hypoglycemic drugs such as metformin, sulfonylureas, and thiazolidinediones, especially in heart transplant patients. Early evidence on newer hypoglycemic agents, including SGLT-2 inhibitors, GLP-1 receptor agonists, and DPP-4 inhibitors, suggests a more favorable risk–benefit profile compared to older drugs. However, further studies are essential to establish their efficacy and safety in NODAT patients [80].

## 6. Complications and Long-Term Consequences

**Figure 3:**
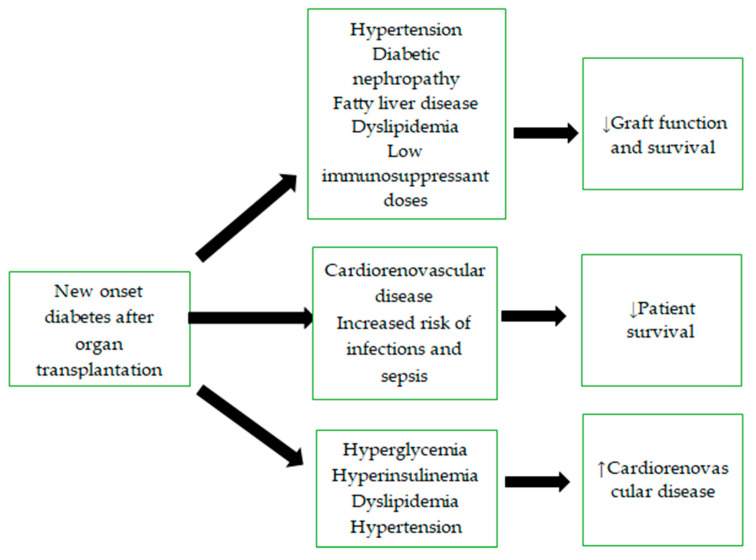
The impact of new onset diabetes after organ transplantation on organ and patient survival (↑ increase, ↓ decrease).

NODAT can lead to usual diabetic complications such as diabetic nephropathy, neuropathy, retinopathy, ketoacidosis, and hypoglycemic episodes. The development of these complications is influenced by factors such as the type of transplanted organ, age, obesity, immunosuppressive therapy, and other cardiovascular risk factors. Patients with NODAT after heart, lung, kidney, and liver transplants experience a higher incidence of graft rejection, infections, and subsequent cardiovascular events, contributing to increased mortality compared to those without diabetes (Figure 3) [8,46,48,95,96].

### 6.1. Influence on the Graft Function

NODAT is a significant complication that adversely affects graft function by increasing the risk of chronic allograft dysfunction, enhancing immune activation and rejection episodes, and by increasing the susceptibility to infections. Hyperglycemia combined with the immunosuppressive therapy increases the risk of opportunistic infections and sepsis-related mortality in transplant patients. Metabolic disturbances in NODAT can heighten immune responses, resulting in increased risk of acute rejection, particularly when the dose of immunosuppressants, such as tacrolimus, is reduced to mitigate the development of NODAT, thereby negatively affecting graft function. Research has shown that kidney transplant recipients with NODAT develop diabetes-related complications earlier, most commonly diabetic nephropathy, which negatively impacts graft function and increases the risk of chronic allograft dysfunction [37,97]. NODAT is also a common complication following liver transplantation, with about a quarter of patients developing NODAT within 10 years of transplantation [38]. A study of 778 liver transplant patients with a median follow-up of 57 months revealed a negative impact of NODAT on graft function and survival. Patients with NODAT had an increased risk of graft loss due to chronic rejection (4.2%) or hepatic artery thrombosis (6.0%) compared to patients without diabetes (chronic rejection 1.3%, hepatic artery thrombosis 3.1%) [98]. In heart transplant patients, diabetes is associated with a higher incidence of acute graft rejection, the development of graft vasculopathy, coronary artery disease, and infections [97]. Additionally, lung transplant patients with NODAT have a significantly higher incidence of acute cytomegalovirus infection and acute graft rejection compared to those without diabetes [99]. Strategies to preserve graft function in patients with NODAT include frequent monitoring of blood glucose levels, which allows early detection and management of NODAT, encouraging patients to engage in regular physical activity and to adopt a healthy diet, adjusting the immunosuppressive regiments to minimize diabetogenic effects, and utilizing antidiabetic medications that have minimal interactions with immunosuppressants.

### 6.2. Influence on Life Expectancy

NODAT is a frequent complication following organ transplantation, significantly affecting patient outcomes by elevating the risk of cardiovascular diseases, infections, allograft dysfunction, and mortality. Kidney transplant patients with NODAT experience significantly shortened survival compared to those without NODAT, with an average reduction in life expectancy of 3 years [6]. A study on kidney and liver transplant recipients demonstrated that NODAT independently increased the incidence of cardiovascular diseases, especially coronary artery disease (HR, 2.46; 95% CI, 1.39 to 4.30), and overall mortality (HR, 1.48; 95% CI, 1.14 to 1.95). These incidences were more prominent in kidney transplants compared to liver transplants [100]. A study involving liver transplant patients also demonstrated higher mortality in those with NODAT compared to patients with normoglycemia or pre-existing diabetes. Patients with NODAT had a significantly higher incidence of infection-related deaths (9.5%) compared to those with pre-existing diabetes (5.0%) or normoglycemia (4.4%). Death due to chronic graft rejection was significantly lower in the normoglycemic group (0.4%) compared to those with NODAT (1.4%) or pre-existing diabetes (1.3%). Ten-year survival rates were 78% for patients with normoglycemia, 75% for those with pre-existing diabetes, and 69% for those with NODAT [98]. Studies on heart transplant patients show mixed results regarding survival. Some studies indicate shortened survival in patients with diabetes compared to those without the condition, while others report similar survival rates in both groups, possibly due to better glycemic control in some studies [97]. The presence of diabetes-related complications, such as cerebrovascular accidents, peripheral vascular disease, kidney failure, and obesity, further reduces survival in heart transplant patients. There is no significant survival difference between those without diabetes (10.1 years) and those with diabetes but no complications (9.3 years). However, survival decreases significantly as the number of complications increases. The average survival for patients with one complication is 6.7 years, and for those with two or more complications, it is 3.6 years [101].

A study involving over 14,000 heart transplant patients found that those with NODAT had worse survival compared to those without NODAT, but there was no significant difference in survival between patients with pre-transplant diabetes and those who developed NODAT [48]. The average survival time for lung transplant patients is 8.3 years, with significant differences based on the presence of diabetes. Patients who had diabetes before transplantation had a shorter average survival time of 6.3 years compared to those who did not have diabetes before transplantation (8.4 years). The difference in survival between patients with NODAT and those who did not develop diabetes was not statistically significant (8.4 vs. 10.4 years, *p* = 0.45). There were no significant differences in the cause of death between patients without diabetes, with NODAT, or with diabetes before transplantation. The incidence of chronic graft rejection at the time of death was similar in all three groups (20%, 16%, and 17%, *p* = 0.83) [96].

Early detection of NODAT, lifestyle modifications, utilizing antidiabetic medications, and adjusting immunosuppressive regiments are crucial for improving patient survival. Additionally, hyperinsulinemia, glucose intolerance, insulin resistance, hypertension, and dyslipidemia further increase the risk of cardiovascular events. Therefore, it is crucial to initiate antihypertensive therapy and antilipemic agents alongside NODAT treatment to help reduce patient mortality. Thiazide diuretics and calcium channel blockers have shown nephroprotective effects in patients receiving calcineurin inhibitors. Statins are beneficial in reducing the risk of developing NODAT, improving insulin resistance, and exerting anti-inflammatory effects. Fluvastatin also offers nephroprotective benefits, in contrast to fibrates, which are nephrotoxic and can reduce the concentration of ciclosporin in heart transplant patients. As such, caution is required when using fibrates in these patients [1,6,24].

### 6.3. Influence on the Chronic Complications of Diabetes

NODAT increases the risk for development of microvascular (retinopathy, nephropathy, and neuropathy) and macrovascular (coronary artery disease, peripheral arterial disease, and stroke) complications. Immunosuppressive therapy, and coexisting conditions, such as obesity, hypertension, dyslipidemia, chronic kidney disease, along with lifestyle factors (smoking, sedentary lifestyle) also have a significant influence on the development of diabetes complications. Patients with a kidney transplant and NODAT develop diabetes complications faster, and the average time required for the development of complications is only 500 days. The most common complications are diabetic nephropathy, which develops in 31.3% of patients and neurological complications, occurring in 16.2% of patients, while diabetic retinopathy (8.3%), ketoacidosis (8.1%), hypoglycemia (7.3%), peripheral vascular disease (4.1%), and hyperosmolarity (3.2%) are less common. The development of renal and neurological complications may be accelerated due to the use of immunosuppressive therapy. It was observed that older age, BMI, non-white race, HCV infection, hypertension, the use of tacrolimus and prolonged cold kidney ischemia (more than 30 h) also have a significant influence. Studies show that NODAT is also associated with an increased risk of developing coronary artery disease, peripheral vascular disease, and cerebrovascular disease [97,102].

Diabetic nephropathy in the transplanted kidney occurs after an average of 5.9 years in patients with pre-transplant diabetes or NODAT. The period from transplantation to the appearance of diabetic nephropathy was longer in patients with NODAT, 9.93 ± 3.07 years, compared to patients with pre-transplant diabetes, 6.68 ± 3.86 years, but the duration of diabetes until the appearance of histological signs of diabetic nephropathy was similar in both groups, 6.68 ± 3.86 compared to 5.90 ± 3.13 years [103]. Pathological changes in diabetic nephropathy are mostly comparable in transplanted and native kidneys. The difference between native and transplanted kidneys is the occurrence of vascular and tubulointerstitial changes caused by acute graft rejection, viral infections, and the nephrotoxic effects of calcineurin inhibitors. More recent studies on the development of diabetic nephropathy in native kidneys indicate the importance of podocytes as the first site of damage with subsequent progression to nodular glomerular sclerosis and interstitial fibrosis. Hyperglycemia leads to the activation of the costimulatory molecule B7-1 in podocytes, which leads to changes in the cytoskeleton and podocyte adhesion, and CTLA-4 antibodies prevent these changes. Belatacept is a newer immunosuppressive drug used in maintenance therapy, and it is a CTLA-4 antibody with a high affinity for B7-1, so there is a possibility that belatacept has a preventive effect on the development of diabetic nephropathy after kidney transplantation, but further research and monitoring are needed to prove this assumption. Recent studies also indicate the importance of the mTOR signaling pathway in podocytes in the progression of glomerular disease, so the use of mTOR inhibitors in immunosuppressive therapy may have a potential role in the prevention of diabetic nephropathy [37,104,105]. A study on kidney transplant patients demonstrated that patients with higher insulin resistance and an IGR (insulin-to-glucose ratio) ≥ 0.092 experienced worse early graft function. Identifying patients with higher insulin resistance and implementing early interventions may enhance graft function [106]. Key strategies for preventing and managing these complications include optimizing glycemic control by adjusting immunosuppressive regiments, regular blood glucose monitoring, and the use of antidiabetic medications. Lifestyle modifications, including smoking cessation, a healthy diet, and regular physical activity, also play a crucial role. Managing coexisting conditions, such as obesity, hypertension, dyslipidemia, and chronic kidney disease, is essential to reduce vascular damage and improve the patients’ quality of life.

## 7. Conclusions

NODAT is a complex and multifactorial complication that often emerges in patients following organ transplantation. Many individuals already have pre-existing risk factors for NODAT prior to transplantation, which can exacerbate the likelihood of developing this condition post-transplant. One of the most significant and modifiable risk factors for NODAT is chronic exposure to immunosuppressive medications, which often have diabetogenic effects. NODAT exacerbates the patient’s vulnerability to infections due to the weakened immune system from both immunosuppressive medications and hyperglycemia. NODAT can lead to the development of traditional diabetes-related complications such as diabetic nephropathy, retinopathy, neuropathy, and cardiovascular issues. These complications not only reduce the patient’s quality of life but also further complicate post-transplant management and therapy.

Effective management involves a combination of lifestyle modifications, pharmacological interventions, careful adjustments to immunosuppressive therapy, and regular monitoring. These approaches aim to prevent complications and address comorbidities, ultimately ensuring better long-term outcomes for transplant patients. Research should focus on the risk–benefit ratio of newer drugs, such as SGLT-2 inhibitors, GLP-1 agonists, and DPP-4 inhibitors, in the context of immunosuppressive therapies and NODAT. NODAT represents a significant challenge for organ transplant recipients due to its multifactorial nature and its interaction with immunosuppressive therapies. While it increases the risk of infection, graft rejection, cardiovascular disease, and diabetes-related complications, early detection, prevention, and treatment strategies can improve patient outcomes. Ultimately, better management strategies will improve patient survival and quality of life.

## Figures and Tables

**Table 1:** Non-modifiable and modifiable risk factors.

Non-Modifiable Risk Factors	Modifiable Risk Factors
- older age - race - genetic predisposition - male gender - deceased donor - positive family history of diabetes - previous corticosteroid therapy - previous glucose intolerance - polycystic kidney disease	- obesity - metabolic syndrome - proteinuria - hypomagnesemia - hepatitis C infection - cytomegalovirus infection - corticosteroids - calcineurin inhibitors - mTOR inhibitors

**Table 2:** Pharmacological treatment of new onset diabetes after organ transplantation.

Hypoglycemic Agent	Advantages	Disadvantages
Metformin	- first choice if GFR > 45 mL/min - no interactions with immunosuppressants - no hypoglycemia - leads to weight loss	- risk of lactic acidosis
Sulfonylurea derivatives and glinides	- strong impact on reducing HbA1c	- risk of hypoglycemia - weight gain - necessity to modify doses according to the level of eGFR - glyburide and glimepiride increase the concentration of ciclosporin - cyclosporine and itraconazole can increase the concentration of repaglinide leading to hypoglycemia
Inhibitors of α-glucosidase		- lack of data
Thiazolidinediones	- no hypoglycemia - safe for patients with chronic kidney disease without dose adjustment - no effect on the metabolism of ciclosporin and tacrolimus	- fluid retention - bone loss and fractures - weight gain
Dipeptidyl peptidase-4 inhibitors	- no hypoglycemia - no weight gain - well tolerated - no interactions with immunosuppressants (except saxagliptin)	- necessity to modify doses of some agents for chronic kidney disease
Glucagon-like peptide 1 (GLP-1) receptor agonists	- weight loss - no hypoglycemia - improved cardiovascular risk - no effect on tacrolimus levels	
Sodium-glucose cotransporter-2 (SGLT2) inhibitors	- weight loss - no hypoglycemia - cardioprotective and renoprotective effects	- urinary tract infections - volume depletion - limited data - necessity to modify doses according to the level of eGFR
Insulin	- can be used in chronic kidney disease or liver dysfunction	- risk of hypoglycemia

## Data Availability

Not applicable.
